# Poster on the wall: My stint as a scientific committee member

**DOI:** 10.51866/mol.711

**Published:** 2024-11-19

**Authors:** Suzane Shiyun Chin

**Affiliations:** 1 MD, FRACGP, Klinik Kesihatan Bandar Maharani, Jalan Hospital, Taman Utama Satu, Muar, Johor, Malaysia. Email: drsuzane.chinshiyun@moh.gov.my

**Keywords:** Scientific conference, Scientific committee, Scientific poster, Research

I returned to work this year in April after a prolonged hiatus, to great furore.

The 26th Family Medicine Scientific Conference (FMSC), a prestigious national event hosted by family medicine specialists from a different state each year, was upon us. This year, my birthplace and current workplace, Johor, played host.

I was immediately inducted into the research team of the scientific committee. Although I had participated in research conferences before, this was my first time serving as an organiser.

It was a novel and memorable experience.

The response to the research competition was overwhelming this year, with over 200 abstracts submitted, reflecting the burgeoning status of primary care centres as focal points for clinical studies.

I quickly realised that the task of a research team committee was no small feat. We conducted abstract reviews, shortlisted entries through group consensus, prepared paperwork and maintained continuous correspondence with authors, judges, IT/logistics staff and board vendors. This correspondence allowed me to bond with many fellow specialists in the state and enabled me to become acquainted with those from other states – connections I likely would not have made without this event.

**Figure 1:**
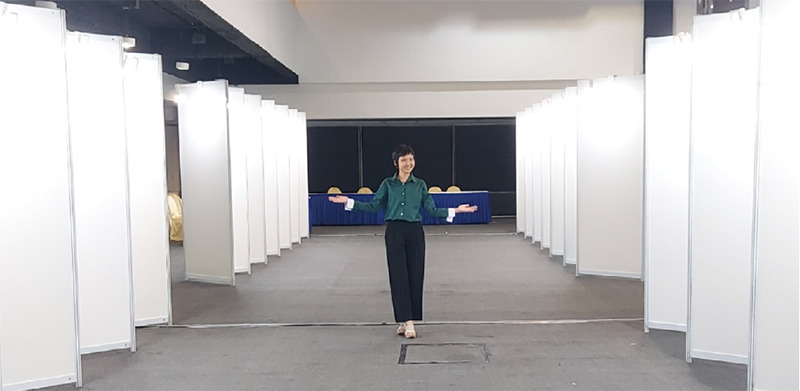

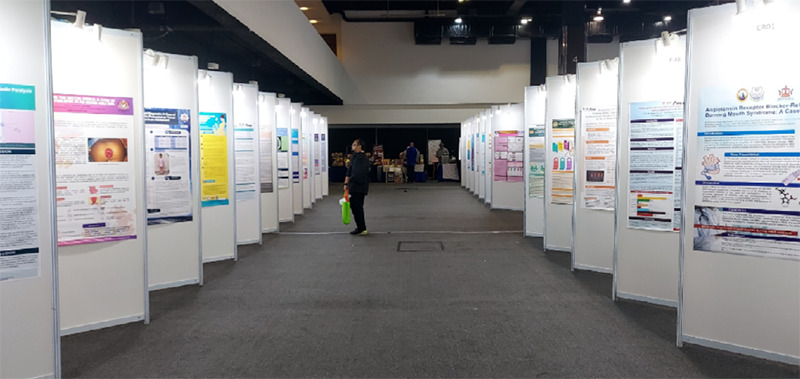
Before and after the poster exhibition in the hall

September arrived in the blink of an eye, and the 26th FMSC kicked off with much pomp and pageantry at the Persada International Convention Centre in Johor Bahru.

Despite the packed schedule, one of the highlights of the conference for me was the unexpected opportunity to speak with the Health Minister, Datuk Seri Dr Haji Dzulkefly bin Ahmad, when he expressed an interest to examine the electronic research poster board. I briefed the minister with much pride on the diverse scope of research conducted at primary care level, which was easily reflected by the unique research entries for the conference.

**Figure 2:**
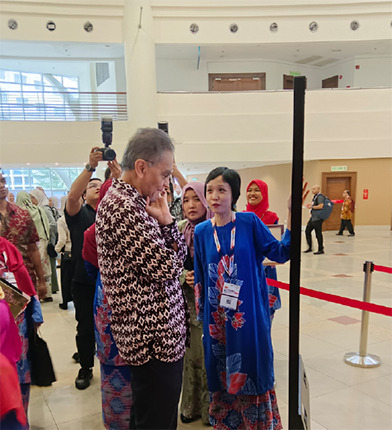
The Health Minister’s visit

Another significant point from my involvement as a research committee member came on the final day of the conference, when we organised a small meeting with the judges to finalise the winners. It was a great learning experience to listen to the input of knowledgeable specialists with years of involvement in clinical research as they dissected each poster for assessment.

**Figure 3:**
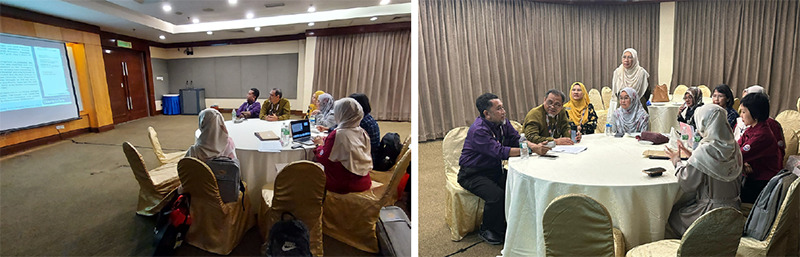
Meeting with the judges to finalise the 26th FMSC poster winners

**Figure 4:**
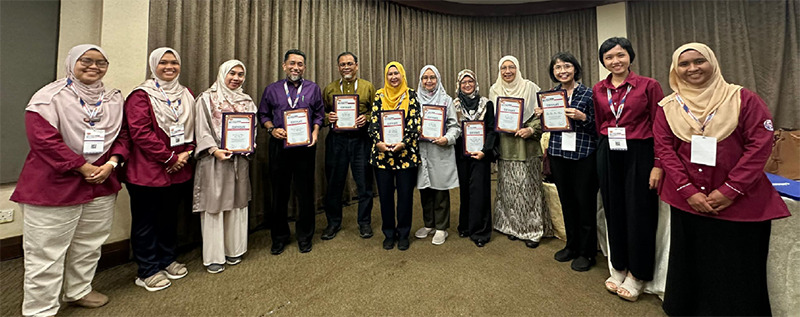
Conclusion of the meeting with the judges of the 26 th FMSC poster competition

Primary care research has come a long way, and I certainly hope to continue to be a part of it indefinitely.

***‘For every minute spent organising, an hour is earned’. — Benjamin Franklin***

While the quote above clearly implies time-saving benefits from proper planning, allow me to put a small spin on Mr Franklin’s words in my context: ‘For every minute spent organising ***an event,*** an hour of ***knowledge or experience will definitely be*** earned’.

We in Johor were extremely happy and proud to host all participants at the 26th FMSC, and we hope everyone came away with a fantastic experience. Congratulations to all participants and winners!

I wish to extend my gratitude to all family medicine specialists and gazettees from Johor (with special mention to Drs Syazwani, Farah, Nasehah, Syafiqah and Hazizul) as well as the guest doctors, the Malaysian Family Medicine Specialists’ Association, exhibitors, vendors and supporting staff, who had worked diligently to ensure the success of the conference.

**Figure 5:**
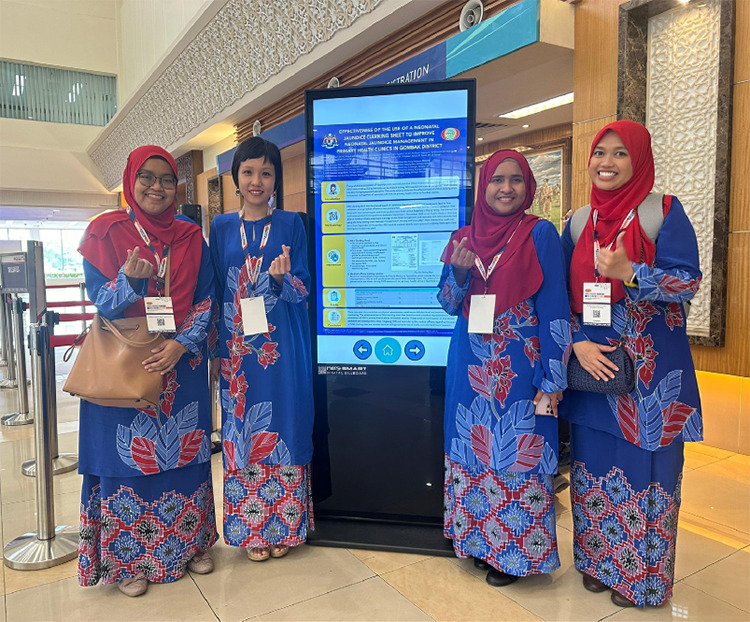
Team members with one of the electronic poster boards

